# A novel, native-format bispecific antibody triggering T-cell killing of B-cells is robustly active in mouse tumor models and cynomolgus monkeys

**DOI:** 10.1038/srep17943

**Published:** 2015-12-11

**Authors:** Eric J. Smith, Kara Olson, Lauric J. Haber, Bindu Varghese, Paurene Duramad, Andrew D. Tustian, Adelekan Oyejide, Jessica R. Kirshner, Lauren Canova, Jayanthi Menon, Jennifer Principio, Douglas MacDonald, Joel Kantrowitz, Nicholas Papadopoulos, Neil Stahl, George D. Yancopoulos, Gavin Thurston, Samuel Davis

**Affiliations:** 1 Regeneron Pharmaceuticals, Tarrytown, New York 10591

## Abstract

Bispecific antibodies, while showing great therapeutic potential, pose formidable challenges with respect to their assembly, stability, immunogenicity, and pharmacodynamics. Here we describe a novel class of bispecific antibodies with native human immunoglobulin format. The design exploits differences in the affinities of the immunoglobulin isotypes for Protein A, allowing efficient large-scale purification. Using this format, we generated a bispecific antibody, REGN1979, targeting the B cell marker, CD20, and the CD3 component of the T cell receptor, which triggers redirected killing of B cells. In mice, this antibody prevented growth of B cell tumors and also caused regression of large established tumors. In cynomolgus monkeys, low doses of REGN1979 caused prolonged depletion of B cells in peripheral blood with a serum half-life of approximately 14 days. Further, the antibody induced a deeper depletion of B cells in lymphoid organs than rituximab. This format has broad applicability for development of clinical bispecific antibodies.

Bispecific antibodies comprise two distinct binding specificities, and can be used in place of two conventional monoclonal antibodies to bind to distinct epitopes. In addition, a bispecific antibody can bridge between its two target proteins and bring them into close proximity. This property offers opportunities for therapeutic applications that cannot be achieved with a mixture of two monospecific antibodies. For example, linking a tumor cell marker with an activating receptor on an effector cell, such as a cytotoxic T cell, can trigger target-dependent tumor cell killing; several such molecules have been approved or are in clinical trials^1^.

Bispecific antibodies have been developed in a variety of different formats. Many employ single chain variable region (scFv) modules, or similar structures that rely on engineered linkers to force the assembly of binding components into the desired configuration. Concerns with many of these formats include a tendency to aggregate, difficulties in production, short serum half-lives, or potential of immunogenicity. Several designs have also been developed in the format of a native antibody, i.e., consisting of two light and two heavy chains. For most of these, the heavy chain Fc-Fc interface is engineered with “knobs” and “holes” or electrostatic charges to actively promote the formation of heterodimers of distinct heavy chains when they are co-expressed^2^,^3^. To avoid heavy-light chain mispairing, a common light chain is typically used that pairs with both heavy chains without altering their respective specificities. Although the presence of the Fc domains can confer the extended serum half-life of conventional antibodies, these strategies still introduce unnatural mutations, and the resulting proteins are potentially immunogenic and unstable. Another native-format design consists of a rat-mouse hybrid^4^, in which there is no mechanism to preferentially promote formation of heterodimers over homodimers. Instead, the difference between the affinities of rat IgG2a and mouse IgG2b for Protein A makes it possible to separate heterodimers from homodimers by selective affinity chromatography. In this format, heavy-light chain mispairing is prevented because these pairings are species-specific. Although a molecule of this type has been approved for clinical use by intraperitoneal injection, it carries the immunogenic profile of rodent proteins in humans.

We sought to devise a format that is free of the disadvantages mentioned above. To avoid engineering the Fc-Fc interface, we adopted the strategy of selective Protein A affinity chromatography, in the context of a fully human antibody. Asymmetry in the ability to bind Protein A is achieved by introducing a local isotype chimera of fully human immunoglobulins, described below, on one of the heavy chains. In addition, a common light chain is utilized.

The ability of bispecific antibodies to trigger redirected T cell killing of tumor cells has been known since 1986^5^. Because of its potential broad utility for treatment of a wide variety of cancers with known cell surface markers, and with the advent of technologies for production of human monoclonal antibodies, this approach has received increasing attention in recent years. The first clinically approved bispecific antibody, catumaxomab, based on the rat-mouse hybrid format, targeted the cell surface marker, EpCAM, for treatment of malignant ascites^6^. A second clinically approved bispecific antibody, blinatumomab, comprising an scFv-based format denoted “Bispecific T-cell Engagers”, targeted the B cell marker, CD19^7^. Many others are currently in development^1^. Because of the great promise of this anti-tumor strategy, we have, as a first application of our format, constructed bispecific antibodies that recognize both the B cell marker, CD20, and the CD3 component of the T cell receptor. We show that they mediate target-dependent lysis of B cells by T cells *in vitro*, possess anti-tumor efficacy in mouse tumor models, and potently deplete B cells in cynomolgus monkeys.

## Results

**Figure 1:**
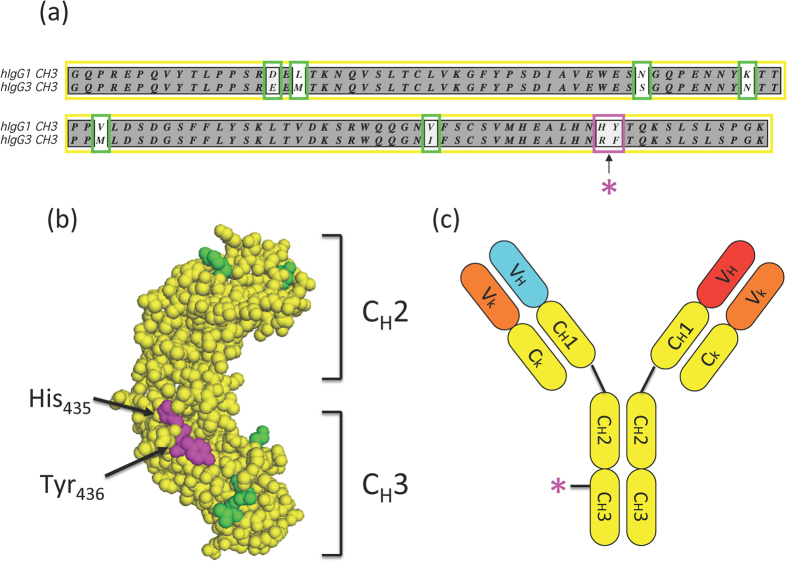
Description of bispecific antibody format. (**a**) Alignment of the sequences of the CH3 domain of human IgG1 and IgG3. (**b**) Localization of the Fc* substitution within the crystal structure of human IgG1. Residues that are conserved between IgG1 and IgG3 are shown in yellow; H_435_ and Y_436_ are shown in magenta; other non-conserved residues are shown in green. (**c**) Schematic diagram of bispecific antibody format. Heavy chain variable regions of different specificity are in red and cyan; common light chain variable regions are in orange.

Unlike other human IgG isotypes, human IgG3 does not bind to Protein A. Previous studies^8^ found that a dipeptide substitution in IgG1, [H_435_R, Y_436_F], ablated the ability of IgG1 to bind Protein A, consistent with co-crystal structural data showing that His_435_ is in direct contact with Protein A^9^. Aligning the sequences of IgG1 and IgG3, we noted that they were identical across a span of 25 residues centered on this dipeptide (Fig. 1a). We reasoned that introducing this dipeptide modification into IgG1, creating a local isotype chimera, would present no new potential T cell epitopes, and consequently likely be non-immunogenic. Additionally, from the known crystal structure of IgG1, it was apparent that His_435_/Tyr_436_, which are surface exposed residues, are spatially embedded within a region that is conserved between IgG1 and IgG3 (Fig. 1b), so the replacement was likely to be sterically compatible. Accordingly, we chose this modification of the IgG1 Fc (for brevity, denoted Fc*), in concert with a requirement for a common light chain, as the basis for our bispecific format (Fig. 1c). A similar approach is applicable to IgG2 and IgG4 as well.

The simplest way of guaranteeing that two antibodies share a common light chain is by engineering mice that can express only a single, “universal” light chain. Because such mice were not yet available for this study, an alternative approach was adopted. Typically, most of the affinity and specificity of antibodies resides in the heavy chain, and it has been reported that substituting an antibody’s cognate light chain with a non-cognate light chain can sometimes maintain the binding specificity of the heavy chain^10^. This led us to use a screening strategy of cross-pairing the heavy and light chains of candidate antibodies to find a light chain that is compatible with both.

To validate our strategy, a CD20xCD3 bispecific antibody was constructed. Panels of anti-CD3 and anti-CD20 human antibodies were generated via immunization of VelocImmune® mice^11^, and light chains from the anti-CD3 antibodies were paired with heavy chains from the anti-CD20 antibodies. The resulting hybrid antibodies were then screened to identify those that retained CD20 binding. In qualitative binding experiments, we observed that many non-cognate light chain pairings reduced or ablated binding to CD20 (Supplementary Table 1). However, some heavy chains were found that were tolerant to many light chain substitutions, and it was possible to select pairings which maintained the binding affinity of the original anti-CD20 antibody (Supplementary Figure 1). Maintenance of cross-reactivity to cynomolgus proteins was also assessed in order to enable potential downstream toxicology studies. Several successful pairings were identified, and one was chosen to generate the bispecific antibody, consisting of the respective anti-CD20 and anti-CD3 heavy chains, combined with the anti-CD3 light chain. The Fc* substitution was introduced on the anti-CD3 heavy chain. These three components were co-expressed in CHO-K1 cells.

**Figure 2:**
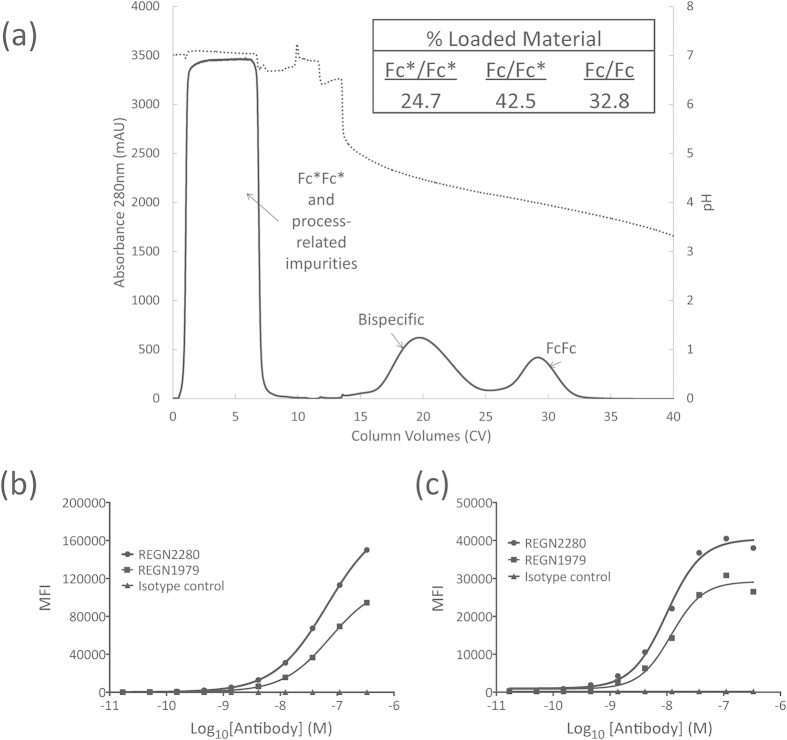
Isolation of bispecific antibody by selective Protein A affinity chromatograpy. (**a**) Chromatogram illustrating the separation of REGN2280 bispecific from the binding (FcFc) and non-binding (Fc*Fc*) homodimers, using a recombinant Protein A resin. The dotted trace shows pH, and the solid trace shows the absorbance at 280 nm of load, wash and gradient elution steps. (**b,c**) Flow-cytometric analysis of CD20xCD3 bispecific antibodies REGN2280 and REGN1979 binding to CD20+/CD3− Raji (**b**) and CD3+/CD20− Jurkat (**c**) cells. Bound antibody is proportional to mean fluorescence intensity (MFI). A non-specific hIgG1 isotype control antibody showed no binding to either cell line.

The resulting mixture of homodimeric and heterodimeric bispecific antibodies was passed over a Protein A affinity column. To avoid potential complications arising from the fact that Protein A has been observed to bind to the variable domains of many members of the V_H_3 family of heavy chain variable domains^12^, a resin with a modified form of Protein A was used that has been shown to have much reduced affinity for these domains^13^ (see Methods). As expected, the homodimeric CD3/CD3 (Fc*/Fc*) species did not bind to the column and flowed through during loading, along with the bulk impurities (Fig. 2a). Bound proteins were then eluted using a pH gradient, and two well separated peaks eluted at pH 4.5 and pH 4.0 (Fig. 2a). Mass spectrometry analysis showed that the first fraction consisted of the bispecific heterodimeric antibody, while the second was the CD20/CD20 (Fc/Fc) parental homodimer. The yield of bispecific antibody was 42.5% of total antibody produced (Fig. 2a), close to the maximum expected yield of 50% resulting from random association of heavy chains. The purified bispecific antibody (denoted REGN2280) could bind to cells expressing CD20 (Fig. 2b) as well as to cells expressing CD3 (Fig. 2c). To address potential issues involving Fc-dependent effector function, a variant of this antibody was also constructed, based on an effector function-minimized version of IgG4 (denoted REGN1979).

**Figure 3:**
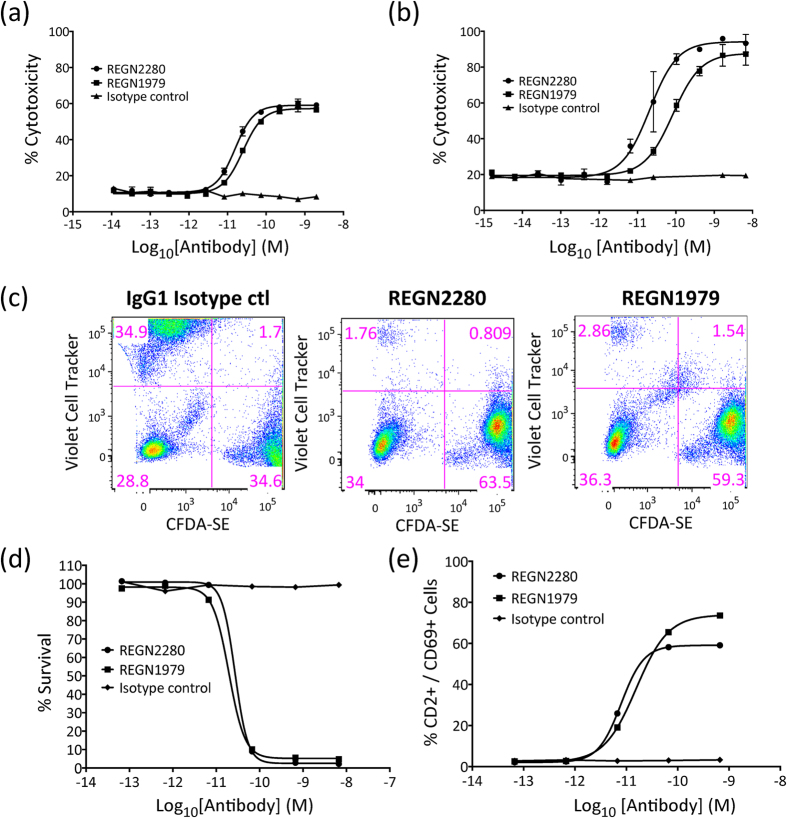
Redirected T cell-mediated cytotoxicity of bispecific antibodies *in vitro*. (**a,b**) Effects of treatment of CD20 positive Raji cells with CD20 x CD3 bispecific antibodies and pre-activated human (**a**) or cynomolgus (**b**) effector lymphocytes (E:T-ratio 10:1). Cell viability was measured by Calcein release assay after 2 hours of incubation. (**c**) Specific lysis of CD20+ target cells upon treatment with 10 ug/ml of CD20 x CD3 bispecific antibodies and effector lymphocytes (E:T ratio 4:1). B16F10.9 cells expressing exogenous CD20 are labeled with Violet cell tracker (upper left quadrants), while control B16F10.9 cells are labeled with CFDA-SE (lower right quadrants). Both REGN2280 and REGN1979 selectively deplete cells expressing CD20. (**d**) Effects of treatment of CD20-positive B16F10.9 cells with CD20 x CD3 bispecific antibodies and effector lymphocytes (E:T ratio 4:1). Cell viability was measured by FACS after 48 hours incubation. (**e**) Dose-dependent effect of the CD20 x CD3 bispecific antibodies on the expression of CD69 on CD2+ lymphocytes in the presence of B16F10.9 expressing CD20 cells after incubation for 48 hours. The surface expression of CD69 on effector cells was detected by flow cytometry using an antigen-specific PE conjugated antibody.

REGN2280 and REGN1979 CD20xCD3 bispecifics were tested in *in vitro* cell killing assays to determine whether they could trigger target-dependent lysis of CD20-positive cells by T cells. In a 2 hour cell cytotoxicity assay, activated T cells of either human or cynomolgus origin were able to lyse CD20-expressing Raji lymphoma cells in the presence of low picomolar antibody concentrations, with EC50 values ranging from 15 to 84pM (Fig. 3a,b). This killing was specific for CD20-expressing cells, because it was not observed when anti-CD3-based bispecific antibodies that targeted non-CD20 antigens were used (data not shown). It also required linking CD20 with CD3, because a mixture of the parental anti-CD20 and anti-CD3 antibodies was ineffective (data not shown). In addition to triggering killing by activated T cells, these antibodies could also induce naïve T cells to kill target cells (Fig. 3c). In this assay, CD20 was exogenously expressed in CD20-negative B16F10.9 cells, designated B16-CD20. These cells were then combined with the parental cells and the mixture was used in a 48 hour cytotoxicity assay. Nearly complete depletion of the CD20-positive population was observed, but not of the CD20-negative population (Fig. 3c,d). Cytotoxicity was also accompanied by induction of the T cell activation marker, CD69 (Fig. 3e). Similar results were obtained with bispecific antibodies of this format constructed with a range of other anti-CD3 and anti-CD20 parental antibodies (data not shown).

**Figure 4:**
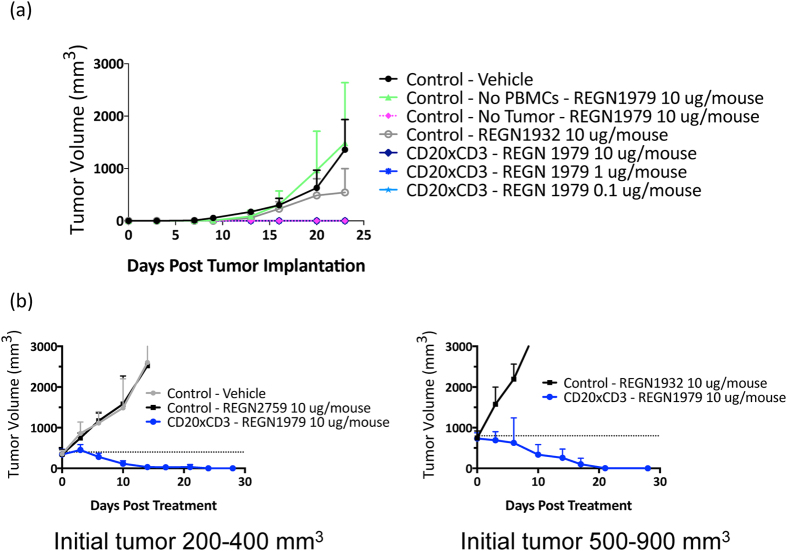
Effects of REGN1979 on tumor growth in *in vivo* mouse tumor models. (**a**) Raji tumor cells were co-implanted with human PBMCs subcutaneously into NSG mice and treated with the indicated amount of REGN1979 or a control antibody via intraperitoneal injection twice a week starting at day 0 for the length of the study. No tumor growth was observed at all dose levels of REGN1979 tested. (**b**) Raji tumor cells were co-implanted with hPBMCs and tumors were allowed to develop until they reached the indicated initial volume (either 200–400 mm^3^ or 500–900 mm^3^). Treatment was then initiated with REGN1979 or a control antibody. These were dosed twice weekly for the duration of the study. Animals dosed with REGN1979 showed complete tumor regression in both initial tumor sizes.

This promising *in vitro* data led us to investigate the activity of the generated CD20xCD3 bispecifics in *in vivo* tumor model systems. For these assays and subsequent studies we chose to focus on REGN1979 to avoid the potentially complicating effects of Fc receptor binding that could arise with REGN2280. Because the anti-CD3 binding arm of REGN1979 does not cross-react with murine CD3, we chose a model system in which human PBMC cells were introduced into immune compromised NOD scid gamma [NSG] mice. Human PBMCs were co-injected with Raji B cell lymphoma cells at a 1:4 ratio subcutaneously (Day 0), and treatment with REGN1979 bispecific was initiated either on day 0, or once tumors had been established (Day 15; 200–400 mm^3^). Immediate treatment of tumor-bearing mice with REGN1979 resulted in complete prevention of tumor outgrowth at all tested drug levels (Fig. 4a), including with doses as low as 1 μg/mouse. For established Raji tumors, twice weekly dosing of with 10 μg/mouse resulted in complete regression of the tumors whereas treatment with either vehicle or control antibody had no effect. Additionally, following tumor regression, tumors did not regrow for at least 30 days after the end of treatment (data not shown). These findings demonstrate that REGN1979 has potent anti-tumor activity *in vivo*.

**Figure 5:**
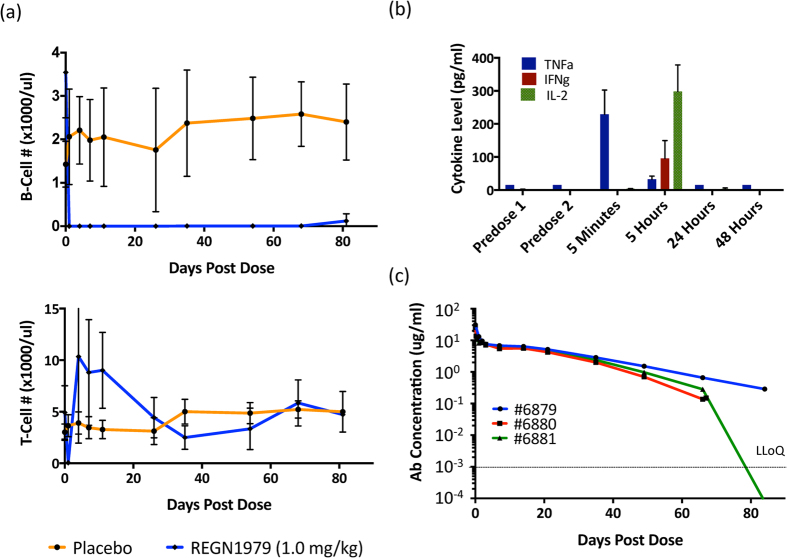
Effects of REGN1979 on circulating B cells and on cytokine levels in cynomolgus monkeys. (**a**) Cynomolgus monkeys were injected with a single dose of REGN1979 (1 mg/kg), and levels of peripheral B and T cells were examined by flow cytometry. Treatment with REGN1979 resulted in complete depletion of measurable circulating B cells for at least 60 days post dose, and a transient loss of circulating T cells. (**b**) Cytokine levels were measured at the indicated time post dose. Compared to pre-dose levels, treatment with REGN1979 resulted in transient increase in all measured cytokines, which returned to base line levels by T = 24 hours. (**c**) Levels of REGN1979 in circulation are shown for the three animals in the cohort (I06879, I06880, I06881). LLoQ represents the lower limit of quantification for the assay.

To determine whether treatment with REGN1979 could deplete circulating B cells in primates, and whether it resulted in any unexpected toxicity, we conducted an exploratory non-GLP pharmacology study in cynomolgus monkeys. Cohorts of three animals were treated with a single i.v. dose of 1 mg/kg of either REGN1979 or placebo, and levels of circulating B and T cells were monitored for 12 weeks. In addition, levels of circulating REGN1979 and inflammatory cytokines were analyzed. Treatment with REGN1979 resulted in an immediate and complete depletion of circulating B cells, persisting for more than 60 days; even by the termination of the experiment on day 82, circulating B cell levels had not fully recovered (Fig. 5a). Levels of circulating T cells were initially also reduced following REGN1979 treatment, but by day 4 post treatment, the number of circulating T cells returned to baseline levels or slightly higher (Fig. 5a). An initial rapid decline followed by rebound of T cells has also been described in human patients treated with blinatumomab, the CD3xCD19 BiTE (Bargou *et al.*^7^). A rapid rise in levels of circulating cytokines was observed following treatment with REGN1979: TNF-α was increased at 5 minutes post dosing, and IFN-γ and IL-2 were also elevated at 5 hours. At 24 hours, all cytokines returned to baseline levels (Fig. 5b). Finally, measurement of circulating levels of REGN1979 showed that it had a calculated serum half-life of 14 days, comparable to the expected half-life of conventional antibodies (Fig. 5c). Monitoring of the animals for the duration of the experiment revealed no unexpected toxicities for REGN1979 at this dose level (data not shown).

**Figure 6:**
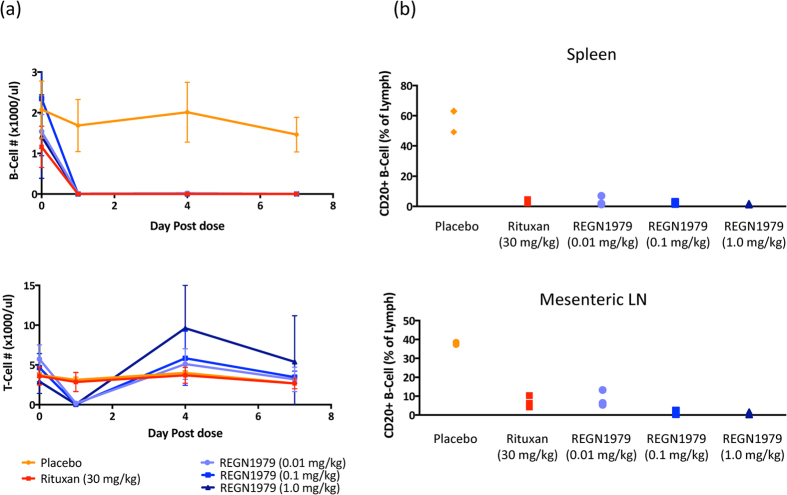
Depth of killing of B cells in peripheral lymphoid tissues of cynomolgus monkeys treated with REGN1979. Cynomolgus monkeys were dosed with the indicated amounts of REGN1979 or rituximab, and levels of circulating B and T cells were analyzed by FACS (**a**). Seven days post dose the animals were sacrificed, and levels of B cells in the peripheral lymphoid tissues were examined by tissue FACS as described in Methods (**b**).

**Figure 7:**
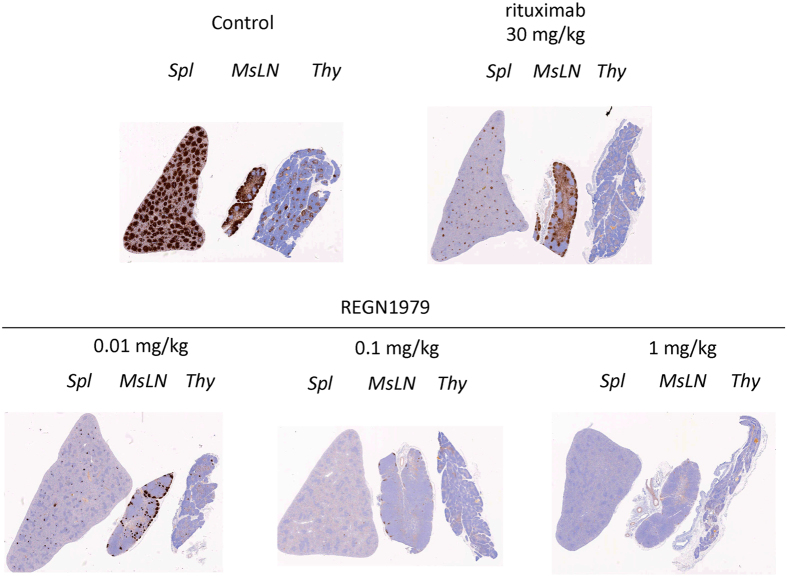
Depth of killing of B cells in peripheral lymphoid tissues of cynomolgus monkeys treated with REGN1979, immunohistochemical analysis. Seven days post dose with the indicated amounts of REGN1979 or rituximab, the animals were sacrificed, and the indicated peripheral lymphoid tissues were stained with anti-CD20 antibodies. Similar results were obtained with antibodies against the B cell markers, CD19 and CD79a (data not shown). Spl, spleen; MsLN, mesenteric lymph nodes; Thy, thymus.

A second experiment was conducted to assess the ability of REGN1979 to deplete B cells from peripheral lymphoid tissues in cynomolgus monkeys. Animals (3 males/group) were treated with a single dose of either placebo, REGN1979, or rituximab by intravenous infusion. Three treatment dose levels of REGN1979, 0.01, 0.1, and 1 mg/kg were studied, as well as a dose of 30 mg/kg of rituximab, comparable to the standard clinical dose of 375 mg/m^2^ 14. Animals were monitored for 7 days and then sacrificed to analyze levels of B cells in peripheral lymphoid organs. Consistent with the results of the initial exploratory study, levels of circulating B cells were depleted from peripheral blood immediately upon treatment, and remained depleted for the course of the study (Fig. 6a). Similarly, animals treated with REGN1979 showed a transient reduction of T cells in peripheral blood, returning to baseline levels by day 4 post treatment (Fig. 6a). The transient reduction of T cells was not observed in animals treated with either placebo or with rituximab. At the conclusion of the study, spleens and lymph nodes were harvested, and B cell levels were analyzed by flow cytometry. In spleen, B cell levels were profoundly decreased to approximately equal levels in all drug treated groups. In mesenteric lymph nodes, B cell depletion by REGN1979 was dose dependent. At 0.01 mg/kg, REGN1979 reduced B cells to similar levels as observed with the 30 mg/kg dose of rituximab. However, at the 0.1 and 1.0 mg/kg doses, REGN1979 reduced B cells to a significantly greater extent that rituximab (Fig. 6b). These findings were similar in other lymph nodes (data not shown). To confirm the flow cytometry findings, tissue sections from spleen, mesenteric lymph nodes, and thymus were analyzed by immunohistochemistry for the presence of B cells. As is shown in Fig. 7, the B cell staining in these tissue sections matches the findings observed in the flow cytometry analysis. Both the 0.1 and 1.0 mg/kg dose of REGN1979 resulted in very few or no detectable B cells in these tissues, whereas residual B cells were readily observable in the tissues from the rituximab treatment group as well as the 0.01 mg/kg REGN1979 group. These findings suggest that CD20xCD3 bispecifics may be more potent at depleting B cells from lymphoid tissues than conventional anti-CD20 antibodies.

## Discussion

We describe a promising new bispecific antibody format and evaluate its capabilities, using a model system that targets CD20 and engages CD3-expressing effector cells. It is possible to construct a fully human heterodimeric bispecific antibody with a common light chain and purify it by selective Protein A chromatography. Additional experiments provide functional validation of this bispecific format: *In vitro,* similar to other CD3-based bispecific antibodies reported in the literature, our bispecific induces efficient activation of T cells and lysis of target cells. *In vivo*, the bispecific antibody REGN1979 shows potent anti-tumor activity in two mouse tumor models. In cynomolgus monkeys, a pharmacologically and physiologically relevant species, the bispecific potently and durably depletes B cells in the circulation as well as in lymph nodes and spleen. Thus, the bispecific format performs well in all stages of this test case, leading to an antibody with potential clinical utility.

The activity of REGN1979 in two mouse tumor models demonstrates that not only does it prevent B cell tumor formation when given at the time of tumor implantation, but also it causes complete regression of large established tumors; the lack of recurrence for at least 30 days, while not fully conclusive, suggests complete eradication. As might be expected for an established tumor, where effector:target ratios are likely to be small and accessibility may be difficult, higher doses were required in the therapeutic treatment model than in the immediate treatment model or in *in vitro* tumor cell lysis assays. It will be of interest to examine the effects of REGN1979 on the markers of regulatory and anergic T cells and cytokine production in these models. It will also be important to assess the activity of REGN1979 in models that do not entail co-injecting tumor cells with T cells or PBMCs, for example, in immunocompetent mice expressing humanized components of CD3.

The ability of REGN1979 to deplete B cells in lymph nodes of cynomolgus monkeys demonstrates that it can be effective not only on circulating B cells but also in less accessible solid tissues. The sharp contrast between the partial depletion observed with conventional doses of rituximab and the essentially full depletion with higher doses of REGN1979 suggests that in a clinical setting, REGN1979 might eradicate malignant cells located in niches where they escape elimination by rituximab, and perhaps greatly reduce the probability of relapse. Furthermore, the long half-life of REGN1979 in cynomolgus monkeys is in distinct contrast to some other formats, which must be administered continuously via either an infusion pump or through frequent injections. In the present studies, we found that just a single dose of REGN1979 depleted B cells for more than 60 days. Levels of REGN1979 much higher than those required for B cell depletion in peripheral blood were maintained for several weeks without apparent toxicity or perturbation of T cells. While it remains to be determined whether the promising results reported here can be achieved in patients, these observations support clinical development of REGN1979^15^.

The bispecific antibody platform introduced here resembles the format and production process of conventional antibodies more closely than others previously discussed in the literature. Production is done using standard mammalian cell culture methods, with overall yields comparable to conventional antibodies. The purification process is straightforward, involving only a minor modification of conventional Protein A purification, and therefore is highly compatible with current standard procedures for large-scale industrial production of conventional monoclonal antibodies. The separation method intrinsically removes homodimers, while formats that rely on engineered interfaces that actively promote heterodimer formation require additional methods to remove homodimeric species that are likely to form as contaminants^16^. However, as with the rat-mouse hybrid bispecific approach, one drawback is that supernatant from our bispecific-producing cells contains approximately 50% of the desired product, the other 50% being comprised of the two forms of homodimers. The production process does not involve reassembly of half-antibodies, which often leads to improperly formed intrachain disulfide bonds^16^. There is also no constraint on the antibody components to carry their affinity and specificity primarily in the light chain, as is the case for “common heavy chain” designs^17^. The final bispecific product of the present format should have all the advantages of native antibodies, including stability, low tendency to aggregate, low immunogenicity, and good pharmacokinetics.

The bispecific antibody format described here represents a versatile platform that provides the means to develop fully human bispecific antibodies in native IgG format, which can readily be produced at large scale for clinical use. It stands out for its simplicity and ease of implementation. We anticipate that these will be useful, not only in the paradigm of T cell-mediated lysis of tumor cells, but also in a broad range of other applications.

## Methods

### Generation of CD3 and CD20 antibodies

Antibodies against human CD3 were generated through immunization of VelocImmune® mice^11^ with the mouse fibroblast cell line MG87 engineered to express human CD3ε and human CD3δ. Mice were given an intraperitoneal injection of 10 million gamma-irradiated cells in a mixture with TiterMax® Gold adjuvant (Sigma-Aldrich). After the 1^st^ injection, mice were boosted two times in a three-week interval with the same number of cells without adjuvant. Mice were given a final boost three days prior to sacrifice. Harvested splenocytes were used to generate hybridomas by fusing with P3 × 63Ag8.653 mouse myeloma cells. The hybridoma culture media were screened for binding specificity to CD3 + Jurkat cells vs CD20- Daudi cells and MG87/CD3 cells vs the parental MG87 cells. Clones the displayed the desirable binding specificity to Jurkat and MG87/CD3 were banked and the heavy and light chain genes were sequenced and cloned into hIgG expression vectors.

Similarly, monoclonal antibodies against human CD20 were generated via immunization of VelocImmune® mice with a DNA plasmid comprising the full-length transmembrane human CD20 gene. The DNA immunogen was administered via an intradermal injection at the basal area followed by electroporation. After the 1^st^ injection, mice were boosted 4 times in a three-week interval with the same DNA injection. Three days prior to sacrifice, mice were boosted with 10 million MG87 cells engineered to express human CD20. The spleens were processed as described above, and the hybridoma culture media were screened for binding to both MG87/CD20 and NSO/CD20 cells vs parental MG87 and NSO cell lines. Clones that displayed the desirable binding specificity to both MG87/CD20 and NSO/CD20 cells were banked and the heavy and light chain genes were sequenced and cloned into hIgG expression vectors.

### Cell Lines and PBMC Isolation

The following cell lines were cultured in RPMI 1640 (Irvine Scientific, USA) medium supplemented with 10% fetal bovine serum (FBS; Tissue Culture Biologicals [TCB], Long Beach, CA), 2 mM L-glutamine, 100 units/ml penicillin, 10 μg/ml streptomycin (Life Technologies, Grand Island, NY): Jurkat, a human T cell lymphoblast-like cell line (DSMZ, Braunschweig, Germany); and Raji, a Burkitt’s lymphoma cell line (ATCC, Rockville, MD). B16F10.9 (ATCC, Rockville, MD) cells were stably transfected with the plasmid pRG984/hCD20, a vector expressing human CD20. The resulting cell line (B16-CD20) was maintained in culture under media conditions described above along with puromycin selection (1 ug/ml). B16-CD20 cells were confirmed for expression of hCD20 by flow cytometry (4-laser BD Fortessa, BD Biosciences, San Jose, CA) using anti-human CD20 clone 2H7 (Biolegend, San Diego, CA). Data were analyzed using Cytobank software.

Human peripheral blood mononuclear cells (PBMC) were isolated from leukapheresis products obtained from normal healthy donors (New York Blood Center, NY, NY) using Ficoll-Paque, (GE Healthcare). Cynomolgus monkey PBMC were isolated from freshly drawn (within 3 hours) heparinized whole blood (Huntingdon Life Science, East Millstone, NJ) using Lympholyte-Mammal Cell Separation Media (Cedarlane, Burlington, Ontario, Canada)

### Protein A Chromatographic separation of bispecific antibody

Lab scale chromatographic separations were performed using an AKTA Avant chromatographic system (GE Healthcare) and a 1.0 cm inner diameter (I.D.) Omnifit Benchmark chromatography columns (Omnifit Ltd) packed with MabSelect SuRe Protein A resin (GE Healthcare) to a 20 cm bed height. After equilibration with two column volumes (CVs) of 20 mM sodium phosphate pH 7.2 the column was loaded to 15 g binding antibody/L with clarified mammalian cell culture fluid. Binding antibody concentration was determined by summation of the bispecific and FcFc homodimer titers. Columns were washed and protein eluted via a pH 6.0–3.0 gradient in 40 mM acetate, 500 mM calcium chloride. Bispecific and FcFc titers were measured using a POROS A 20 μm column (2.1 mm × 30 mm, 0.1 mL) cat#2-1001-00, and Fc*Fc* titers were measured by loading the flowthrough over a POROS G 20 μm column (2.1 mm × 30 mm, 0.1 mL) cat#2-1002-00.

### Cell binding of purified CD20xCD3 bispecific antibody

Binding of CD20 × CD3 bispecific antibodies to CD3 and CD20 expressing cells was determined by flow cytometry. Briefly, 2 × 10^5^ Jurkat (CD3+/CD20−) or Raji (CD20+/CD3−) cells were incubated for 30 minutes at 4 °C with serial dilutions of CD20 × CD3 bispecific or hIgG1 isotype control antibodies. After washing twice with cold PBS supplemented with 1% FBS (wash buffer), cell surface bound antibody was detected by incubating the cells with phycoerythrin (PE)-labeled anti-human IgG antibody for 30 minutes at 4 °C. Cells were washed twice in the same buffer and the geometric mean fluorescence (MFI) of stained cells was measured using a FACSCanto II cytometer (BD Biosciences, San Jose, CA, USA) with Diva software (BD). Cell populations were visualized on forward scatter vs side scatter and were gated to exclude dead cells. Wells containing no antibody or secondary only were used to establish background fluorescence. Four-parameter non-linear regression analysis was used to obtain EC_50_ values for cell binding using Prism software (GraphPad Software, La Jolla, CA).

### *In Vitro* Cytotoxity assays

The efficacy of the CD20XCD3 bispecific antibodies to mediate tumor cell lysis by pre-activated human or cynomolgus T cells was determined in a 2 hour calcein release assay^18^. In brief, isolated human or cynomolgus PBMCs were activated with T cell activation beads specific for human (Human T-Activator CD3/CD28 Beads, Life Science) or cynomolgus (T Cell Activation/Expansion Kit, non-human primates, Miltenyi Biotec) T cells. Cells and activation beads were culture for 7 (human) and 21 (cynomolgus) days in complete media (RPMI supplemented with 10% FBS, 100 U/mL penicillin, 100 μg/mL streptomycin, 2mM L-glutamine) containing recombinant human IL-2 (30U/mL for human, 100 U/mL for cynomolgus). Cynomolgus cells were restimulated at day 14 with fresh activation beads, and maintained in complete media with 100 U/mL rhIL-2. On the day of the assay, CD20 expressing Raji cells (2 × 10^6^ cells/mL) were labeled with 8 μM Calcein-AM for 30 minutes at 37 °C and washed 3 times with media. Calcein-labeled target cells (10,000–20,000 cells/well) were plated in 200 μL with activated T cells (effector/target cell ratio 10:1) and serial dilution of bispecific antibodies or IgG1 isotype control in complete media for 2 hours at 37 °C. Following incubation, the plates were centrifuged and supernatants were transferred to a translucent black clear bottom plate for fluorescence analysis. Percent cytotoxicity was calculated using the equation:


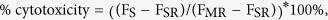


where F_S_ is calcein release from the test well, F_SR_ is spontaneous calcein release and F_MR_ is maximal calcein release from cells lysed by Triton-X. Results are expressed as the % specific lysis (mean ± SD) from duplicate wells.

A FACS based approach was used to determine the ability of the CD20xCD3 bispecific antibodies to mediate specific tumor cell lysis by naïve T cells. To track the loss of target bearing tumor cells relative to non-target bearing tumor cells, B16-CD20 or parental B16F10.9 cells were labeled with 1μM Violet Cell tracker (Life Sciences) or 1μM CFDA-SE (Life Sciences) respectively. After labeling, the two cell types were counted, mixed at a 1:1 ratio, and 2.5 × 10^5^ total cells were plated in 96-well flat bottom plates for overnight culture at 37 °C. Separately, isolated human PBMC were plated in complete RPMI media at 1 × 10^6^ ml and incubated overnight at 37 °C in order to enrich T cells. The following day, 5 × 10^5^ of T cell enriched naïve PBMC were added to the plated target cells (with a 4:1 ratio of Effector to B16-CD20 Target cells) along with serially diluted antibodies, and co-incubated for 48 hours at 37 °C.

After 48 hours, effector and labeled target cells were then removed from cell culture plates using an enzyme free cell dissociation buffer, and analyzed by flow cytometry.

For FACS analysis, cells were incubated with directly conjugated αCD2 and αCD69 Abs for evaluation of T cell activation, and stained with a dead/live far red cell tracker for evaluation of specificity of killing.

EC50 of % cytotoxicity and T-cell activation were determined using Prism. Values were calculated using 4-parameter non-linear regression analysis.

### Animal Studies

Female NOD *scid* gamma (also known as NOD-*scid* IL2Rgamma^null^ (NSG)) mice (Jackson Laboratories, Bar Harbor, Maine) were approximately 12-week-old at the start of studies. The scid mutation and loss of the IL2 receptor gamma chain in these mice leads to a deficiency in mature B cells, T cells, and NK cells allowing for engraftment of human hematopoietic stem cells^19^ or PBMCs^20^. For these experiments, human PBMCs were obtained from a single healthy donor (Reach Bio, Seattle, WA); this donor was positive for HLA-DR3 in order to match Raji cells. Cells were frozen and stored in liquid nitrogen and thawed immediately before use. All experiments performed were approved by Regeneron’s Institutional Animal Care and Use Committee (IACUC), and were carried out in accordance with the approved guidelines. For all tumor studies, mice were weighed and tumor growth was measured twice a week using calipers. Tumor volume was estimated as ½ (length × width^2^).

### Immediate Treatment Model

Raji cells (2 × 10^6^) were admixed and co-implanted with hPBMCs (0.5 × 10^6^) subcutaneously into the right flank of female NSG mice on Day 0. Mice were treated with REGN1979 intraperitoneally (i.p.) twice a week starting on Day 0 for the length of the study. Control mice were treated with a non-binding antibody control, REGN1932, or with vehicle alone. (n = 5 mice per group).

### Therapeutic Treatment Model

Raji cells (2 × 10^6^) were admixed and co-implanted with hPBMCs (0.5 × 10^6^) subcutaneously into the right flank of female NSG mice on Day 0. On Day 15, when tumors were approximately 200–400 mm^3^, mice were randomized into treatment groups (Table 2; *n* = 5 per group) and dosed twice per week i.p. with REGN1979, a non-binding control mAb REGN2759, or vehicle alone for the duration of the study. For all studies, mice were weighed and tumor growth was measured twice a week using calipers. Tumor volume was estimated as 1/2(length × width^2^).

### Statistical Analyses

Statistical analyses were performed utilizing GraphPad software Prism 5.0 (MacIntosh Version). Statistical significance for tumor size between treatment groups was determined by two-way ANOVA with Tukey’s multiple comparisons post-test. Data from each of the readouts were compared across treatment groups. A threshold of p < 0.05 was considered statistically significant, as indicated by symbols (*, @, #). Mice that died prior to the end of study were removed from the combined tumor growth curve (but not the individual mouse growth curve) graphs as indicated and statistical analysis in order to analyze by two-way ANOVA. Non-statistically significant interactions are noted as NS.

### Cynomolgus Monkey Studies

#### Animals

Male cynomolgus monkeys were obtained from Covance Laboratories. Monkeys were 3–4 years of age (3.1 to 4.1 kg). Animals were housed in stainless steel cages equipped with a stainless steel mesh floor. Environmental controls were set to maintain the following animal room conditions: temperature range of 20 to 26 °C, relative humidity range of 30 to 70%, 10 or greater air changes/hour, and a 12 hour light/12-hour dark cycle. The light/dark cycle was interrupted for study related activities. Animals were given various cage-enrichment devices and fruit, vegetable, or dietary enrichment. Animals were commingled in accordance with Covance standard operating procedures. Animals were offered Certified Primate Diet #2055C (Harlan Laboratories, Inc.) one to two times daily unless fasted for study procedures and water was supplied to animals *ad libitum.* Veterinary care was available throughout the course of the study and animals were examined by the veterinary staff as warranted by clinical signs or other changes. All procedures in the protocol were in compliance with applicable animal welfare acts and were approved by the local Institutional Animal Care and Use Committee (IACUC), and were carried out in accordance with the approved guidelines.

#### Experimental design

Experiment 1: Following randomization into study groups, animals (3 males per cohort) received a single intravenous infusion of placebo or REGN1979 1 mg/kg and animals were observed out to 12 weeks. REGN1979 was in an aqueous buffered vehicle, pH 5.8 containing 10 mM histidine, 10% (w/v) sucrose, and 0.1% (w/v) polysorbate 80 provided at a nominal protein concentration of 25.0 mg/mL

Experiment 2: Following randomization into study groups, animals (3 males per cohort) received a single intravenous infusion of rituximab at 30 mg/kg or REGN1979 at 0.01, 0.1, or 1 mg/kg. REGN1979 was in an aqueous buffered vehicle, pH 5.8 containing 10 mM histidine, 10% (w/v) sucrose, and 0.1% (w/v) polysorbate 80 provided at a nominal protein concentration of 25.0 mg/mL. Rituximab was dosed as supplied by manufacturer at a concentration of 20 mg/mL. Blood samples were collected and analyzed for routine hematology, clinical chemistry and coagulation parameters. Animals were fasted for at least 8 h for clinical chemistry. At study termination, all animals were subjected to a complete necropsy evaluation. Weights were recorded for a comprehensive panel of organs, and representative samples of tissues were collected and preserved in 10% buffered formalin. As immunopharmacology was a focus for these studies, blood was collected for cytokine analysis and blood and tissue were collected for immunophenotyping (IPT).

#### Peripheral blood immunophenotyping

Blood samples (∼1 ml) were periodically collected in tubes containing the anticoagulant potassium EDTA for peripheral blood immunophenotyping by flow cytometry for B lymphocytes (CD45+/CD20+); T lymphocytes (CD45 + CD3+);. Samples were analyzed with a FACSCanto II Flow Cytometer (BD Biosciences) and DIVA 6.0 software. Monoclonal antibodies used for the staining of cells were anti-CD45 (PerCP; clone D058-1283; BD Biosciences), anti-CD20 (APC-Cy7; clone L27; BD Biosciences), and anti-CD3 (FITC; clone SP34; BD Biosciences).

During sample analysis, total lymphocyte populations were identified on the flow cytometer using a heterogeneous lymphocyte gating strategy consisting of CD45 fluorescent staining and side-scatter characteristics (SSC) demarcation (CD45_high_SSC_low_) to delineate lymphocyte populations. Additionally, a dual-platform methodology was used to enumerate the absolute values for each phenotype. Relative values for each phenotype obtained from the flow cytometer were multiplied by the absolute lymphocyte count from the hematology analysis to enumerate absolute cell counts.

#### Tissue Immunophenotyping

At the scheduled necropsy, samples of the mesenteric lymph node (half of the right node), and thymus (anterior portion) were collected from all animals. Approximately 0.5 g of spleen and thymus were also collected. Following macroscopic examination and organ weights (as appropriate), spleen and thymus samples were weighed and placed into a media-containing tube. Single cell suspensions of each tissue were prepared, and total cellularity was determined for each spleen and thymus sample. Equivalent numbers of cells were stained with antibodies to enumerate phenotypes. Heterogeneous lymphocyte gating strategy of CD45 fluorescent staining and side-scatter characteristics demarcation was used to delineate lymphocyte populations on the flow cytometer. B lymphocytes (CD45+/CD20+) were quantitated using flow cytometry Immunophenotyping results were enumerated as percent relative (%).

#### H&E and Immunohistochemistry (IHC)

Tissues from each animal were embedded in paraffin and sectioned at 5 microns. Sections were placed on slides, processed for routine histology and stained with hematoxylin and eosin. In addition, lymphoid tissue sections from spleen, mesenteric lymph node and thymus were stained to detect B cells with anti-CD20 antibodies (Clone EP459Y, Abcam), Anti-CD19 (Clone LE-CD19, Novus Biological), or Anti-CD79a (Genetex #GTX2056), using standard IHC techniques.

#### Cytokine Analysis

Blood samples were collected into tubes containing potassium (K3) EDTA as the anticoagulant and maintained on a chilled cryorack before and after centrifugation; samples were centrifuged within 1 hour of collection and plasma was harvested. Plasma was analyzed for levels of IFN-γ, IL-2, and TNF-α.

#### Pharmacokinetics

Concentrations of total REGN1979 in monkey serum were measured using an enzyme-linked immunosorbent assay (ELISA). The lower limit of quantitation (LLOQ) is 0.00313 μg/mL in neat monkey serum. The ELISA method employs a microtiter plate coated with REGN654, a mouse anti-human IgG, CH1 domain specific monoclonal antibody, and utilizes REGN1979 as a standard. REGN1979 and placebo were used to prepare the reference standard and quality control (QC) (REGN1979 only) samples. The standards, controls, and samples were added to the microplate, and REGN1979 captured on the plate was detected using biotinylated REGN1548 followed by NeutrAvidin conjugated with horseradish peroxidase. REGN1548 is a mouse anti-human Fc monoclonal antibody that binds to Fc* but not to unmodified Fc. A luminol-based substrate specific for peroxidase was then added to achieve a signal intensity that is proportional to the concentration of total REGN1979.

## Additional Information

**How to cite this article**: Smith, E. J. *et al.* A novel, native-format bispecific antibody triggering T-cell killing of B-cells is robustly active in mouse tumor models and cynomolgus monkeys. *Sci. Rep.* **5**, 17943; doi: 10.1038/srep17943 (2015).

## Supplementary Material

Supplementary Information
