# Supplementary material for: A novel, native-format bispecific antibody triggering T-cell killing of B-cells is robustly active in mouse tumor models and cynomolgus monkeys
Source: Sci Rep. 2015 Dec 11;5:17943. doi: 10.1038/srep17943 (PMC4675964; doi:10.1038/srep17943)

# A novel, native-format bispecific antibody triggering T-cell killing of B-cells is robustly active in mouse tumor models and cynomolgus monkeys

Eric J. Smith, Kara Olson, Lauric J. Haber, Bindu Varghese, Paurene Duramad, Andrew D. Tustian, Adelekan Oyejide, Jessica R. Kirshner, Lauren Canova, Jayanthi Menon, Jennifer Principio, Douglas MacDonald, Joel Kantrowitz, Nicholas Papadopoulos, Neil Stahl, George D. Yancopoulos, Gavin Thurston, and Samuel Davis

**Supplementary Information**

Supplementary Table 1

A panel of anti-CD20 heavy chains were co-expressed with a panel of anti-CD3 light chains, and supernatants from transient transfections were evaluated for binding to Raji (CD20+) cells by flow cytometry. Antibodies showing at least a 3-fold signal above background binding to control CD20-negative cells (Jurkat, HEK293) were scored as +, while those showing lower levels of binding were scored +/-, and those showing no binding above background were scored as negative (NT = not tested). We observed that several heavy chains (CD20-1 and CD20-3) retained the ability to bind CD20 when paired with most of the non-cognate light chains tested, and we therefore selected these heavy chains for further characterization.

Supplementary Figure 1

The CD20-1 /CD3-11 heavy chain/light chain pair identified as described in Supplementary Table 1 was purified and compared to the purified parental anti- CD20 antibody by flow cytometry, as described in Methods. This non-cognate heavy/light chain pairing demonstrated similar binding affinity to Raji (CD20+) cells by FACS when compared to the parental. As expected, no binding was observed with the parental anti-CD3 antibody. The CD20-1 heavy chain, combined with the CD3-11 heavy and light chains, were therefore chosen to generate a CD20xCD3 bispecific.

Supplementary Methods

*Characterization of CD20 Heavy Chain/CD3 Light Chain hybrid antibodies*

To generate hybrid antibodies, CHO-K1 cells were transiently transfected with a ratio of 3:1 light chain to heavy chain plasmid DNA, using standard Lipofectamine protocols. After culturing for an additional 72-96 hr, supernatants were collected.

To assess binding to CD20, 20 ul of supernatant was incubated with cells (Raji, Jurkat or HEK293 cells) for 45 minutes at $4^0$C. The cells were then washed, incubated with the secondary anti-Human PE for an additional 30 minutes at $4^0$C, washed again and then resuspended for analysis. The geometric mean fluorescence (MFI) of stained cells was measured using a BD FACS Canto II flow cytometer.

# Supplementary Table 1

| | | anti_CD20 heavy chain | | | | | | | | | | | |
|---|---|---|---|---|---|---|---|---|---|---|---|---|---|
| | | **CD20-1** | **CD20-2** | **CD20-3** | **CD20-4** | **CD20-5** | **CD20-6** | **CD20-7** | **CD20-8** | **CD20-9** | **CD20-10** | **CD20-11** | **CD20-12** |
| anti_CD3 light chain | **CD3-1** | + | + | + | + | + | + | - | + | + | + | - | - |
| | **CD3-2** | + | + | + | + | + | - | - | - | + | +/- | + | - |
| | **CD3-3** | + | - | + | +/- | - | +/- | - | - | - | +/- | + | - |
| | **CD3-4** | + | - | + | - | - | +/- | - | - | - | NT | - | - |
| | **CD3-5** | + | + | + | + | - | - | - | - | + | + | + | - |
| | **CD3-6** | + | - | + | +/- | - | +/- | - | - | - | NT | - | - |
| | **CD3-7** | + | + | + | + | + | + | - | + | + | +/- | + | - |
| | **CD3-8** | + | - | + | - | - | +/- | - | - | - | - | - | - |
| | **CD3-9** | + | + | + | + | + | + | - | + | + | + | +/- | - |
| | **CD3-10** | + | + | + | + | + | + | - | + | + | + | + | - |
| | **CD3-11** | + | + | + | + | + | + | - | + | + | + | - | - |
| | **CD3-12** | - | - | - | - | - | - | - | - | NT | NT | - | - |
| | **CD3-13** | + | - | - | - | +/- | +/- | - | - | NT | NT | - | - |
| | **CD3-14** | + | - | + | + | - | +/- | - | - | NT | NT | - | - |
| | **CD3-15** | + | - | - | - | - | - | - | - | NT | NT | - | - |

## Supplementary Figure 1

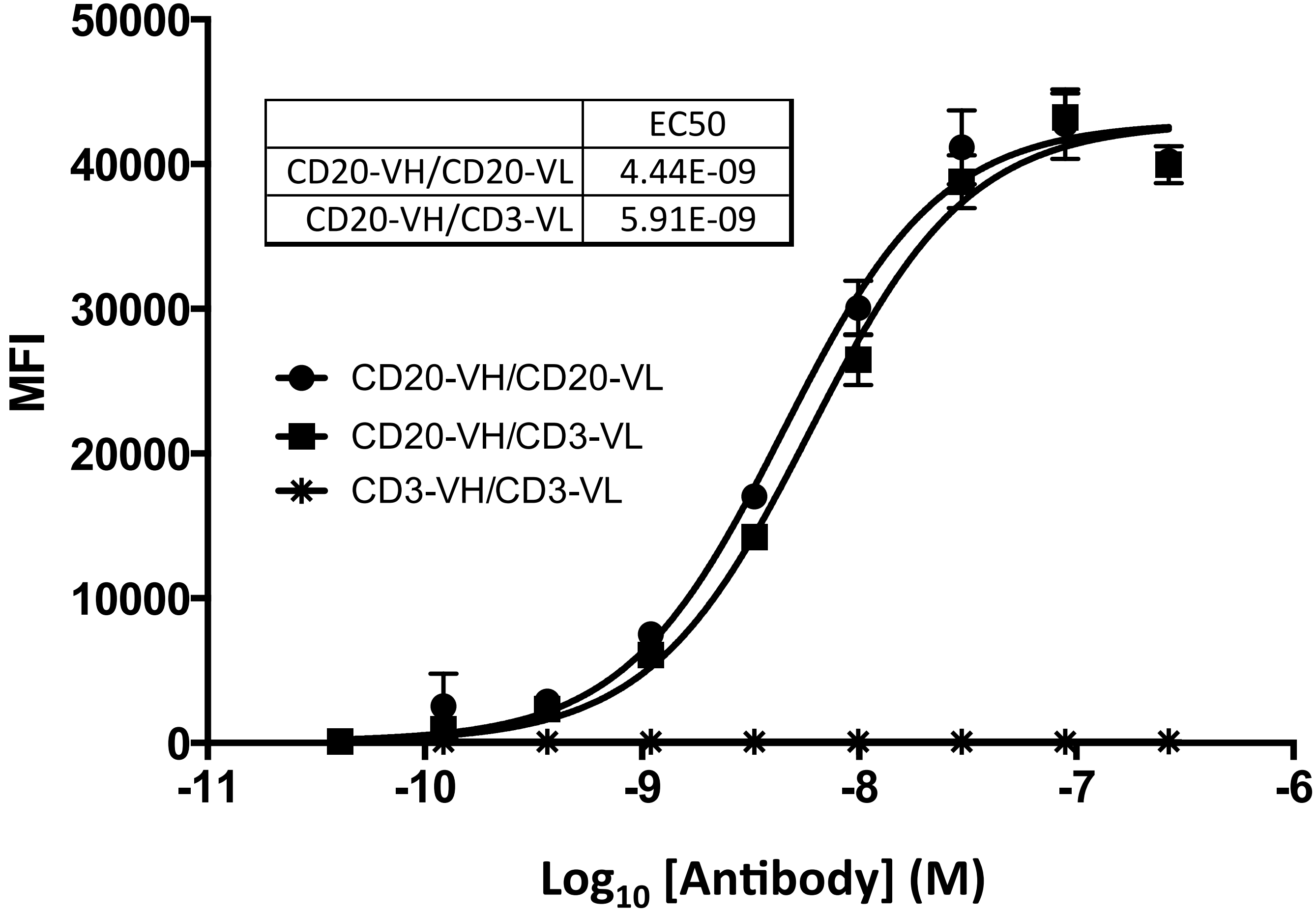

Supplement: Supplementary Information [file srep17943-s1.pdf]
